# Hemispheric Lateralization Interrupted: Material-Specific Memory Deficits in Temporal Lobe Epilepsy

**DOI:** 10.3389/fnhum.2013.00546

**Published:** 2013-09-02

**Authors:** Kim Celone Willment, Alexandra Golby

**Affiliations:** ^1^Department of Neurology, Brigham and Women’s Hospital, Boston, MA, USA; ^2^Department of Psychiatry, Brigham and Women’s Hospital, Boston, MA, USA; ^3^Golby Lab, A Surgical Brain Mapping Laboratory, Department of Neurosurgery, Brigham and Women’s Hospital, Boston, MA, USA

**Keywords:** material-specific, memory, hemispheric lateralization, temporal lobe epilepsy, fMRI

## Abstract

The hemispheric lateralization of memory has largely been informed through the study of patients with temporal lobe epilepsy originating from medial temporal sources (mTLE). The material-specific model of memory relies on the basic framework that the left temporal lobe mediates verbal memories, while the right temporal lobe mediates non-verbal memories. Over the years, this model has been refined, and even challenged, as our understanding of the material-specific memory deficits in mTLE has been further elaborated in the neuropsychological and neuroimaging literature. The first goal of this mini-review is to highlight the major findings in the mTLE literature that have advanced and expanded our understanding of material-specific memory deficits in mTLE. Second, we will review how functional neuroimaging patterns of material-specific hemispheric lateralization in mTLE are being translated into the innovative clinical application of preoperative fMRI memory mapping.

## Introduction

Temporal lobe epilepsy (TLE) is the most common form of localization-related epilepsy in adults, accounting for approximately 60% of cases (Hauser et al., 1991). The most common site of epileptogenesis in TLE originates from medial temporal lobe (MTL) structures, including the hippocampus and anterior subregions (including anterior parahippocampal cortex) (McMillan et al., 1987; Meencke and Veith, 1992; Wolf et al., 1993; Blume, 2006). Medial TLE (mTLE) is often poorly controlled with medications and class I evidence supports the use of surgical resections, specifically anterior temporal lobe resections (ATLR). Both the underlying disease focus and surgical management of mTLE can lead to significant disruption of function within regions and networks that are critically involved in memory function (Fisher et al., 2000; Lee et al., 2002; Sabsevitz et al., 2003; Stroup et al., 2003; Gleissner et al., 2004; Baxendale et al., 2006; Lineweaver et al., 2006; Binder et al., 2008; Hermann and Seidenberg, 2008). The study of memory deficits resulting from the underlying pathology and surgical management of mTLE has been the foundation for much of our understanding regarding memory function (Scoville and Milner, 1957; Penfield and Milner, 1958; Milner, 1970). One of the most influential frameworks describes the hemispheric specialization of memory based on verbal and non-verbal characteristics. The material-specific model dates back to Brenda Milner’s observation that post-operative lesions in the left MTL resulted in abnormalities in verbal memory, while right temporal lobe lesions caused reductions in memory for non-verbal material (assuming left cerebral language dominance) (Milner, 1966). Based on the foundation of material-specific memory deficits, our understanding of hemispheric lateralization of memory function has expanded over time (for a review, see Saling, 2009) and continues to be a heuristic employed in clinical practice and surgical decision making in mTLE (Baxendale, 2008). In fact, material-specific fMRI patterns of hemispheric asymmetry are currently being investigated as a means to estimate the risk for memory decline following ATLR in mTLE. Unlike other reviews on the material-specific memory in mTLE, we focus that later part of this mini-review on the translation of these imaging methods to preoperative clinical mapping.

**Table:** 

**MATERIAL-SPECIFIC MEMORY MODEL**
Hemispheric specialization of memory, such that verbal learning and memory is more dependent on dominant (usually left) hemisphere medial temporal lobe structures, and visuospatial learning is more dependent on medial temporal structures within the non-dominant hemisphere
**PRESURGICAL DECISION MAKING**
The decision to pursue surgical options (lesionectomy, lobectomy, etc.) is based on information gathered regarding: clinical history, electroencephalographic (EEG) data, neuroimaging data (MRI, PET, MEG), neuropsychological testing, and psychosocial functioning
**SURGICAL MANAGEMENT OF FOCAL EPILEPSIES**
The goal of epilepsy surgery is to identify an area of abnormal cortex from which seizures originate and remove it without causing damage to surrounding functioning tissue

A number of neuropsychological and neuroimaging studies have demonstrated a relationship between material-specific memory impairment and lateralized MTL dysfunction in mTLE (Jones-Gotman, 1986; Seidenberg et al., 1996; Helmstaedter et al., 1997, 2003; Jones-Gotman et al., 1997, 2010; Gleissner et al., 1998; Pillon et al., 1999; Golby et al., 2002; Powell et al., 2005). However, inconsistencies have also been reported (for a review, see Saling, 2009) and our understanding of the functional specialization of temporal regions in material-specific memory and the participation of these functionally specialized nodes in a larger network framework of memory processes is still evolving. The variability in the material-specific findings in mTLE has also broadened our understanding of mTLE as a network disease that can affect the function of or connections between any number of nodes that are critical for memory. For instance, material-specific memory deficits in pre- and post-operative mTLE can result from focal dysfunction in subregions within the MTL, including the hippocampus and parahippocampal and entorhinal cortices, dysfunction within the lateral temporal lobe, and disconnections between MTL and ipsilateral cortical connections. mTLE has also been associated with patterns of disrupted functional localization and lateralization resulting from functional reorganization or compensatory regional involvement (Adcock et al., 2003; Thivard et al., 2005), which has allowed for the investigation of the functional implications of neuroplastic changes.

## Material-Specific Memory Deficits and mTLE

The link between verbal memory deficits and the language-dominant mTLE is one of the most consistent patterns of material-specific memory deficits in mTLE (Jones-Gotman et al., 2000), although variability in verbal memory findings can be elicited by accounting for certain task-specific factors (Saling et al., 2002; Saling, 2009). In the non-verbal domain, evidence for a specific link between the right temporal lobe and spatial memory is considerably weaker. Some have argued that visual-spatial memory is a bilateral process, which may largely be mediated by the verbalizability of the information to be learned (van Asselen et al., 2006). To-date, pre- and post-operative ATLR studies using neuropsychological and neuroimaging techniques have produced a myriad of inconclusive data that support non-lateralized findings, including measures of navigation (Maguire et al., 1996; Jokeit et al., 2001), maze learning (Bohbot et al., 1998; Astur et al., 2002), scene recognition (Maguire and Cipolotti, 1998; Spiers et al., 2001a), plan drawing (Spiers et al., 2001b), abstract design (Piguet et al., 1994; Dige and Wik, 2001), and faces (Hermann et al., 1997; Reminger et al., 2004). Furthermore, a number of standard tests of visuospatial memory, including the Rey–Osterrieth Complex Figure, commonly used in evaluation of mTLE, do not reliably distinguish between left and right mTLE (Lee et al., 2002; McConley et al., 2008).

Studies focused on understanding the inconsistencies in the patterns of material-specific memory deficits in mTLE have found evidence for functional dissociations based on the variable influence of extratemporal, intratemporal, and intra-MTL regional dysfunction and highlight the larger network context that can be disrupted by mTLE pathology and surgical management.

### Extratemporal cortical influences

Extratemporal neocortical and connectivity abnormalities in mTLE have been reported both contralateral and ipsilateral to the side of seizure onset (Oyegbile et al., 2004; McDonald et al., 2008; Mueller et al., 2010; Ji et al., 2013). Below we discuss three examples of extratemporal influence over material-specific findings in unilateral mTLE.

#### Frontal lobe language functions

In cases of language-dominant mTLE, extent of hemispheric disruption has the potential to impact language functioning or adequacy (Hermann et al., 1988, 1992). It has been suggested that the strong connections between the inferior frontal cortex and the MTL make frontal lobe language functions particularly sensitive to MTL pathology (Powell et al., 2004). In fact, diffusion tensor imaging (DTI) evidence for reduced left hemisphere language connections in patients with left mTLE compared to both controls and right mTLE patients has been reported (Powell et al., 2007). Functionally, language adequacy has been shown to act as a significant confound when lateralizing verbal memory encoding (Hermann et al., 1988, 1992; Saling et al., 2002). That is, when controlling for language adequacy on a word-list learning task, performance does not correspond with side of seizure focus (right or left) (Hermann et al., 1988, 1992; Saling et al., 2002). Therefore, list-learning performance appears to be less specific to unilateral MTL pathology, and instead corresponds more strongly to extratemporal language processes. Interestingly, retention of verbal information over a delay period has been shown to be less influenced by language adequacy making it a stronger marker of MTL function in left mTLE (Hermann et al., 1988, 1992; Saling et al., 2002).

#### Frontal lobe memory functions

In addition to material-specific patterns of hemispheric asymmetry, neuroimaging studies have found evidence for hemispheric asymmetries within the frontal lobes that corresponds to the component stages of memory (encoding and retrieval) (for reviews, see Desgranges et al., 1998; Cabeza and Nyberg, 2000; Lepage et al., 2000; Fletcher and Henson, 2001). The “hemispheric encoding and retrieval asymmetry” (HERA) model argues that there are observable hemispheric differences according to the stage of memory processing – encoding being preferentially associated with left frontal regions, and memory retrieval showing greater right-sided frontal activation regardless of material type (Desgranges et al., 1998; McDermott et al., 1999; Cabeza and Nyberg, 2000; Lepage et al., 2000; Nyberg et al., 2000; Fletcher and Henson, 2001; Grady et al., 2001; Johnson et al., 2003). Kennepohl et al. (2007) reported convincing evidence within the MTL that aligns with the HERA pattern – the left entorhinal cortex was significantly more active than the right regardless of material type during encoding trials. They found less convincing evidence for the lateralization of retrieval to the right MTL. The work of Kennepohl et al. (2007) raises the possibility that some variability associated with material-specific memory deficits in mTLE could be accounted for by other factors related to hemispheric asymmetries. Future studies in mTLE could be designed to take the HERA pattern into account to further deconstruct the functional specialization of MTL regions and their relationship to post-operative memory outcomes.

#### Parietal and striatal contributions

The neuroimaging literature suggests that a consistent network of brain regions participates in spatial navigation, including the hippocampus proper, the parietal lobe, striatum, occipitotemporal regions, cingulate cortex, and parahippocampal cortices (Aguirre et al., 1996, 1998; Maguire, 1997; Maguire et al., 1998; Jokeit et al., 2001). Two basic strategies in spatial navigation have been described – egocentric and allocentric (Iaria et al., 2003). Allocentric navigation strategies use landmarks and the spatial relationship to a target in order to create a visual map of the environment. Animal and human studies have found support to link allocentric navigation to MTL structures, including the hippocampus and parahippocampal cortex (O’Keefe and Dostrovsky, 1971; Morris et al., 1982; Bohbot et al., 1998, 2004). Egocentric navigation, which describes determining the position of targets in an environment relative to one’s body or position, has been localized to regions within the parietal lobe and striatum in animals and humans (Wiener, 1993; Weniger et al., 2009). Therefore, task-specific features and the extent of potential extratemporal mTLE-related pathology, mainly along MTL-parietal and MTL-striatal connections, may account for variability of visuospatial memory in mTLE.

Spatial navigation studies have also shown patterns of dynamic interaction between right and left temporal regions, and both left and right mTLE patients have demonstrated impairment (Maguire et al., 1996; Astur et al., 2002; Glikmann-Johnston et al., 2008; Canovas et al., 2011). Similarly, measures of object-location have found evidence for bilateral temporal contributions (Baxendale et al., 1998; Incisa della Rochetta et al., 2004; Kessels et al., 2004; Stepankova et al., 2004); although, most studies suggest that the ability to navigate, learn, and recall arbitrarily related objects and locations is right lateralized (Abrahams et al., 1997, 1999; Bohbot et al., 1998; Johnsrude et al., 1999; Duzel et al., 2003; Crane and Milner, 2005; Parslow et al., 2005; Diaz-Asper et al., 2006; Piekema et al., 2006). Overall, the potential bilateral nature of spatial memory may limit the detection of material-specific patterns, particularly during spatial navigation tasks.

### Intratemporal organization

Medial and lateral divisions of the temporal lobes are variably affected by mTLE and surgical resections (Fisher et al., 2000; Lee et al., 2002; Sabsevitz et al., 2003; Stroup et al., 2003; Gleissner et al., 2004; Baxendale et al., 2006; Lineweaver et al., 2006; Binder et al., 2008; Hermann and Seidenberg, 2008) and can influence the nature and extent of memory deficits. Below we discuss the findings related to the underlying semantic structure of verbal memory tasks and contributions of task-specific factors to material-specific verbal memory deficits in mTLE.

#### Semantic-arbitrary distinction

Performance on verbal memory tasks consistently varies relative to the degree the task draws on pre-existing semantic associations (Saling et al., 2002; Saling, 2009). Verbal paired-associate learning studies have shown that patients with language-dominant seizure foci are typically impaired when it comes to learning arbitrary word-pairs. This is known as the semantic-arbitrary distinction and is also supported by studies of story memory (Rausch and Babb, 1987; Saling et al., 1993). However, language-dominant MTL seizure foci do not reliably interfere with learning semantically related word-pairs (Saling et al., 1993, 2002). Similarly, both patients with right and left mTLE tend to be mildly impaired on story memory tasks, which are rich in semantic context (Saling et al., 1993, 2002). Findings related to the semantic-arbitrary distinction also extend to list-learning tasks that allow for semantic clustering (Helmstaedter et al., 1997; Saling et al., 2002), such that the presence of a semantic clustering component may mask a performance deficit on a list-learning task and interfere with the detection of memory impairment linked to underlying language-dominant MTL dysfunction.

In contrast, arbitrary paired-associate learning has been correlated with imaging findings in left mesial temporal structures, specifically the perirhinal cortex (Lillywhite et al., 2007) and the arbitrary-semantic effect has been confirmed in left mTLE patients following standard *en bloc* resections (Helmstaedter and Elger, 1996; Helmstaedter et al., 1997; Saling et al., 2002). More specifically, a lateral to medial gradient has been reported, such that medial structures have been shown to play a critical role in forming arbitrary associations, but are not necessary if semantic associations are pre-existing (Saling et al., 2002). In the pre-existing semantic conditions, cortical or lateral regions are recruited due to their involvement in housing semantic stores (Lillywhite et al., 2007; Saling, 2009).

### Intra-MTL regional specialization

The exact nature of the discrete functional contributions of MTL structures to memory processes remains a matter of intense debate, particularly within the literature on component processes of recognition memory (Aggleton and Brown, 2006; Eichenbaum et al., 2007, 2012; Squire et al., 2007; Henke, 2010). Below we discuss the predominant models of intra-MTL functional specialization during encoding and recognition.

#### Item-in-context model of encoding

Based on the collection of findings from the recollection and familiarity literature (for reviews, see Davachi, 2006; Diana et al., 2007; Eichenbaum et al., 2007), Eichenbaum et al. (2012) proposed a model of episodic memory encoding or “item-in-context” memory. Work in both animals and humans, argues that cortical inputs along the ventral “what” stream first process information regarding objects and events in perirhinal and lateral entorhinal regions, while information regarding spatial context from the dorsal “where” stream is processed by the medial entorhinal and parahippocampal cortex (Suzuki and Amaral, 1994; Furtak et al., 2007; Kerr et al., 2007; van Strien et al., 2009; Wang et al., 2011). Encoding of episodic memories is then proposed to involve the binding or convergence of object and context information from these regions within the hippocampus. Retrieval of episodic memories when cued by either an object or context would reactivate the bound representation within the hippocampus and retrieve the corresponding item or context information by reactivating the “what” and “where” streams (Eichenbaum et al., 2012). The item-in-context model of encoding is consistent with the idea of a medial-lateral gradient of temporal lobe functional specialization discussed above, wherein medial structures are necessary to form novel or arbitrary associations.

#### Recollection and familiarity

Most agree that recognition memory consists of two processes: recollection and familiarity. Recollection refers to the recognition of a stimulus that is bound to contextual details, while familiarity describes an awareness of a stimulus in the absence of contextual details from the original encoding episode (for reviews, see Yonelinas and Levy, 2002; Skinner and Fernandes, 2007). The dual process model suggests that these two processes are mediated by distinct MTL systems; with recollection linked to hippocampal function and familiarity associated with the perirhinal cortex (Aggleton and Brown, 1999, 2006; Yonelinas and Levy, 2002; Eichenbaum et al., 2007). The study of surgical mTLE patients has made a strong case for the dual process model. In fact, Bowles et al. (2007, 2010) have demonstrated a double dissociation, wherein standard ATLR mTLE patients exhibited isolated impairments in recollection and sparing of familiarity processes, while patient NB, who underwent a resection localized to the perirhinal and entorhinal cortices, displayed a selective familiarity impairment (Bowles et al., 2007, 2010). A number of functional imaging studies have also reported that activation of the hippocampus occurs with episodic recollection and memory for associations and context, whereas activation of the perirhinal and parahippocampal cortex is linked with familiarity (for reviews, see Davachi, 2006; Diana et al., 2007; Eichenbaum et al., 2007). Bowles et al. (2010) also showed that overall recognition performance was comparable between the ATLR patients and patient NB, which provides evidence against the main counterargument to the dual process model that states the functional specialization of MTL regions is primarily related to memory strength (Squire et al., 2004, 2007; Wais et al., 2008).

## fMRI Memory Mapping for Preoperative Prediction of Memory Outcomes

Mapping brain memory patterns using Blood oxygen dependent (BOLD) fMRI has been gaining momentum in clinical practice as a means to estimate the risk for memory decline following ATLR in mTLE. The foundation for clinical fMRI mapping is based on the patterns of material-specific findings discussed above, as well as patterns of neuroplasticity and reorganization and their relationship to outcome measures. While there are still methodological and even material-specific challenges (Jansen et al., 2009; Binder, 2011), preoperative fMRI memory mapping has the potential to be a promising addition to the current methods of outcome prediction and as a non-invasive alternative to the intracarotid amytal test (IAT).

**Table:** 

**ESTIMATING POTENTIAL FUNCTIONAL DEFICITS**
Neuropsychological testing, the intracarotid amytal test (IAT), and electrocorticography are the current gold standard for determining whether cognitive or functional decline might be anticipated following ATLR

Neuroimaging studies in mTLE have revealed functionally asymmetric patterns of activation within MTL structures contralateral to the seizure foci during material-specific encoding (Golby et al., 2002; Powell et al., 2007; Banks et al., 2012; Alessio et al., 2013; Sidhu et al., 2013). Functional asymmetries have been associated with variability in neural reorganization within different populations of mTLE patients (Mechanic-Hamilton et al., 2009), with greater asymmetries in mTLE patients with an early age of onset compared to patients with later onset (corresponding to the literature on language lateralization in mTLE). However, it is unclear how well patterns of contralateral activation reflect compensatory activation, as performance levels do not always equate between control subjects and patients (Richardson et al., 2006; Powell et al., 2007). As a result, some have argued that contralateral activation is more appropriately regarded as a marker of network disruption (Powell et al., 2007).

Predictive studies of memory outcomes following ATLR have generally relied on two perspectives to guide decision making (Chelune et al., 1991; Chelune, 1995). The functional reserve hypothesis proposes that the level of functioning of the hippocampus contralateral to the seizure focus will determine the memory outcome. In contrast, the functional adequacy model predicts that post-operative memory outcome will be inversely related to the level of preoperative functioning of the tissue to be resected (Chelune, 1995; Chelune and Najm, 2000).

The functional adequacy model is supported by findings that demonstrate mTLE patients with better preoperative memory functioning are at greater risk for significant memory declines, than patients with low average or poor preoperative memory functioning (Chelune et al., 1991; Hermann et al., 1995; Helmstaedter and Elger, 1996; Jokeit et al., 1997; Davies et al., 1998; Stroup et al., 2003; Gleissner et al., 2004; Baxendale et al., 2006, 2007; Lineweaver et al., 2006; Bonelli et al., 2010). fMRI studies have also been supportive of the functional adequacy model, suggesting that patients with greater ipsilateral activation compared to contralateral MTL activation have greater memory decline following temporal lobectomy (Binder et al., 2008; Frings et al., 2008; Powell et al., 2008; Bonelli et al., 2010; Dupont et al., 2010). Interestingly, a recent DTI study has also shown that stronger connectivity between a default mode network node in the posterior cingulate cortex and the epileptogenic hippocampus, compared to the contralateral hippocampus, has also been linked to greater post-operative memory decline, suggesting a broader network adequacy model (McCormick et al., 2013).

The functional reserve hypothesis has received less support; however, recent neuroimaging work has found evidence for a more nuanced understanding of the functional reserve hypothesis based on anterior and posterior divisions of the MTL (Bonelli et al., 2010). Support for the functional adequacy model was found in patterns of anterior MTL activation, such that the greater asymmetry toward the left was associated with greater decline in verbal memory (Bonelli et al., 2010). However, support for a functional reserve hypothesis was found in patterns of activation in the posterior MTL ROI such that greater asymmetry toward the left was associated *less* verbal memory decline after ATLR (Bonelli et al., 2010). The authors interpreted the posterior findings as intrahemispheric or intra-MTL reorganization of anterior MTL function. Recent data also suggests that the functional reserve and adequacy models may apply differently in the context of right or left MTL seizure foci: memory outcomes for patients with left mTLE may be best predicted by well-functioning right MTL, while patients with right mTLE might be more dependent on the extent of remaining memory function with ipsilateral MTL structures (Banks et al., 2012).

## Summary

The underlying focal pathology and surgical management of mTLE has informed much of our understanding about the material-specific lateralization of memory function. As research methodologies and cognitive theories have been developed, their application to the study of memory deficits in mTLE has refined and even challenged the model of material-specificity. Within the verbal domain, recent work has highlighted the importance of accounting for language adequacy and pre-existing semantic associations. The association between non-verbal deficits and right mTLE has been less consistent than the link between verbal memory and left mTLE, but tasks such as object-location binding have demonstrated promise. Finally, mTLE patients demonstrate aberrant lateralization of activation patterns on functional neuroimaging studies during material-specific memory tasks. These patterns are now being investigated as potential tools for clinical mapping to estimate post-operative memory outcomes following ATLRs.

## Conflict of Interest Statement

The authors declare that the research was conducted in the absence of any commercial or financial relationships that could be construed as a potential conflict of interest.
